# P-2064. The presence of learning difficulties is associated with higher disease burden in patients diagnosed with influenza: a real-world survey in an older population

**DOI:** 10.1093/ofid/ofaf695.2228

**Published:** 2026-01-11

**Authors:** Abid A Kabir, Annabelle Nicholson, James Lucas, James Piercy, Fritha Hennessy

**Affiliations:** Adelphi Real World, Bollington, England, United Kingdom; Adelphi Real World, Bollington, England, United Kingdom; Adelphi Real World, Bollington, England, United Kingdom; Adelphi Real World, Bollington, United Kingdom, Bollington, England, United Kingdom; Adelphi Real World, Bollington, United Kingdom, Bollington, England, United Kingdom

## Abstract

**Background:**

Although the clinical burden of influenza in older individuals is well-documented, limited data exist characterising this burden in individuals with learning difficulties (LD). We aimed to describe the incremental real-world impact of influenza on the quality of life (QoL) in older individuals (aged ≥60 years) associated with LD.

**Figure 1:**
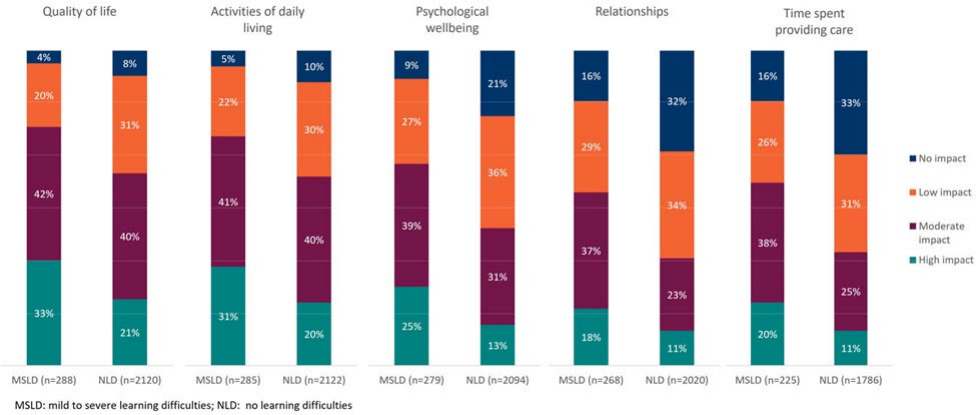
Physician-stated impact of influenza on aspects of life

**Figure 2:**
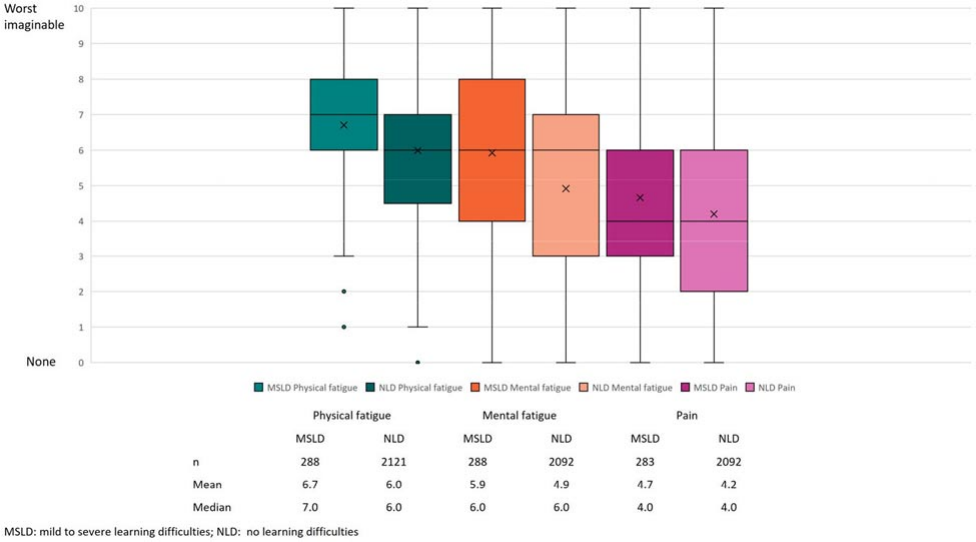
Physician-stated severity of fatigue and pain

**Methods:**

Data were drawn from the Adelphi Influenza Disease Specific Programme™, a cross-sectional survey with retrospective data elements of physicians and patients with influenza in France, Germany, Italy, Spain, the United Kingdom and the United States collected between January 2024-July 2024. Healthcare professionals (HCPs) reported data on demographics, clinical characteristics, and symptoms for 2-4 consecutively consulting patients that had acute influenza. HCPs also provided information regarding their perception of disease burden and QoL. Analyses were descriptive.

**Results:**

Overall, 961 HCPs reported data for 2457 patients, of whom 301 had mild to severe learning difficulties (MSLD) and 2156 had no learning difficulties (NLD). For MSLD patients, mean (SD) age was 78.7 (8.6) years and 53.2% were male. For NLD this was 71.6 (8.2) and 51.7%. In total, 6% of MSLD and 23% of NLD patients were employed. 89% of MSLD and 77% of NLD had concomitant conditions; the most common were chronic pulmonary disease (MSLD: 31%, NLD: 30%), hypertension (MSLD: 36%, NLD: 20%) and diabetes (MSLD: 19%, NLD: 20%). At data collection, 25% of MSLD and 15% of NLD had severe/critical influenza.

Caregiver support was required by 71% of MSLD and 38% of NLD patients, with 30% and 9%, respectively, living in a long-term care facility. Just over half had been vaccinated against influenza (MSLD: 57%, NLD: 56%)

Influenza had a high impact on patient’s psychological wellbeing (MSLD: 25%, NLD: 13%), QoL (33%, 21%) and activities of daily living (31%, 20%; Figure 1). HCPs scored MSLD physical fatigue 6.7, mental fatigue 5.9 and pain 4.7 (NLD: 6.0, 4.9, 4.2 respectively; Figure 2).

**Conclusion:**

Patients with MSLD had a high disease burden due to their influenza, which impacted psychological wellbeing, QoL and daily living. Ongoing research including the patient perspective would inform the implementation of targeted care for older adults with LD.

**Disclosures:**

All Authors: No reported disclosures

